# Drug-induced subacute cutaneous lupus erythematosus associated with docetaxel chemotherapy: a case report

**DOI:** 10.1186/1756-0500-7-785

**Published:** 2014-11-05

**Authors:** Noelle Y Wong, Laurie M Parsons, Martin J Trotter, Roger Y Tsang

**Affiliations:** Faculty of Medicine, University of Calgary, Calgary, AB Canada; Department of Medicine, Division of Dermatology, University of Calgary, Calgary, AB Canada; Department of Pathology and Laboratory Medicine, University of Calgary, Calgary, AB Canada; Department of Oncology, Division of Medical Oncology, University of Calgary, Tom Baker Cancer Centre, 1331 29 St NW, Calgary, Alberta T2N 4N2 Canada

**Keywords:** Drug-induced subacute cutaneous lupus erythematosus, SCLE, Docetaxel, Chemotherapy, Drug reaction, Breast cancer, Sjögren’s syndrome

## Abstract

**Background:**

Drug-induced subacute cutaneous lupus erythematosus is an uncommon disorder associated with the use of pharmacological agents including systemic chemotherapy.

**Case presentation:**

We report a case of docetaxel-induced subacute cutaneous lupus erythematosus in a 60-year-old Caucasian female with Sjögren’s syndrome diagnosed 2 months after receiving docetaxel as part of the adjuvant FEC-D (5-fluorouracil, epirubicin, cyclophosphamide, docetaxel) chemotherapy protocol for early stage breast cancer. Although the exact mechanisms behind the autoimmune response elicited by docetaxel are unclear, the involvement of anti-SSA/Ro antibodies has been implicated.

**Conclusion:**

This case highlights the symptom severity and clinical course of docetaxel-induced subacute cutaneous lupus erythematosus, and highlights the importance of recognizing this uncommon but potentially severe chemotherapy-associated cutaneous reaction.

## Background

Subacute cutaneous lupus erythematosus (SCLE) is a type of cutaneous lupus erythematosus, most commonly characterized by photodistributed, non-scarring, papulosquamous or polycyclic annular plaques. SCLE is often associated with high titers of anti-SSA/Ro antibodies in the serum, and roughly 20% of newly diagnosed cases of SCLE are attributed to a drug or another exacerbating agent [1]. Development of drug-induced SCLE has been linked to a number of pharmacological agents including thiazide diuretics, calcium channel blockers, angiotensin converting enzyme inhibitors, and taxanes [2, 3]. Taxanes, such as docetaxel and paclitaxel, belong to the anti-microtubule class of chemotherapeutic agents and are commonly used in the systemic treatment of non-small cell lung cancer, ovarian cancer, breast cancer, and many other solid tumor malignancies [3]. In this case report, we describe a case of docetaxel-induced SCLE in a 60 year old female with Sjögren’s syndrome diagnosed 2 months after receiving docetaxel as part of the adjuvant FEC-D (5-fluorouracil, epirubicin, cyclophosphamide, docetaxel) chemotherapy protocol for early stage breast cancer, and highlight its severity, clinical course, and importance in recognizing this uncommon chemotherapy-associated cutaneous reaction.

## Case presentation

**Figure 1:**
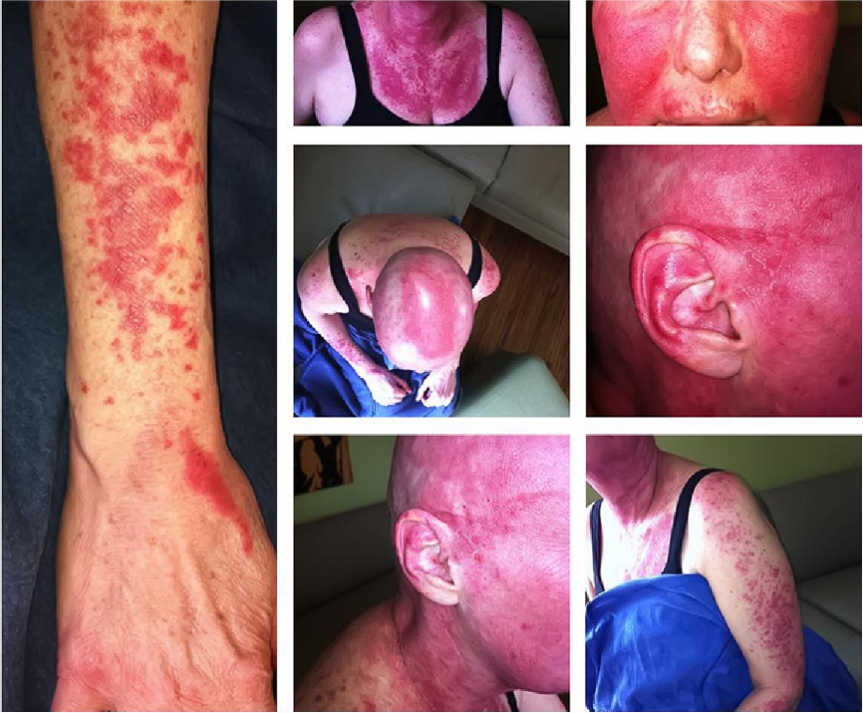
**Severe erythematous desquamating rash.** The rash involved the forearms, anterior trunk, scalp, cheeks, ears, neck, back, as well as membranes of the nose and vagina resulting in epistaxis and vaginal bleeding.

**Figure 2:**
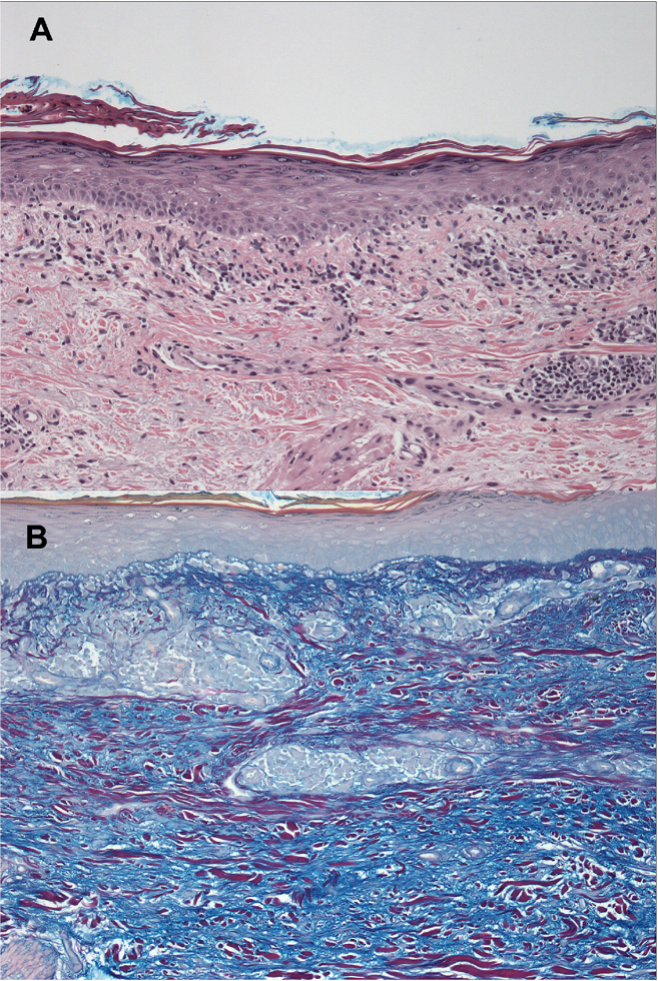
**Photomicrographs of a skin biopsy.** Photomicrographs show an interface dermatitis with associated mid-dermal peri-vascular lymphocytic inflammation (**A**: hematoxylin and eosin, 100X) and dermal mucin deposition (**B**: Hale’s colloidal iron, 100X).

A 60-year-old post-menopausal Caucasian woman with Stage IIB (T2 N1) invasive ductal carcinoma of the left breast commenced adjuvant chemotherapy with the FEC-D (5-fluorouracil, epirubicin, cyclophosphamide for 3 cycles, followed by docetaxel for 3 cycles) protocol with G-CSF support (peg-filgrastim) after undergoing breast-conserving surgery and axillary lymph node dissection. Her past medical history is significant for chronic benign neutropenia and Sjögren’s syndrome primarily manifested by dry eyes, myalgias, and arthralgias, without a pre-existing history of lupus or other connective tissue disorders. She reports no known drug allergies aside from a rash from adhesive tapes, and her medication profile consists of duloxetine, hydroxyquinine, and celecoxib. Two days following the administration of the first cycle of FEC chemotherapy, she developed an exanthema described as a mild erythematous maculopapular pruritic rash on her extensor forearms and anterior trunk, which was felt to be characteristic of a typical chemotherapy-associated rash, given its near complete resolution prior to her subsequent chemotherapy cycle. Nonetheless, the rash did recur with subsequent cycles of FEC chemotherapy, and the switchover to docetaxel after 3 cycles of FEC chemotherapy was delayed by 1 week to allow for its improvement with supportive measures including anti-histamines and a topical corticosteroid. The decision was then made to begin her docetaxel treatment with standard dexamethasone pre-medication. Shortly after the first cycle of docetaxel, however, she developed a moderately severe, erythematous desquamating rash initially located on her forearms and anterior trunk. The severity and extent of this rash differed from that seen with FEC. With the second cycle of docetaxel, despite the addition of a course of prednisone, the rash progressed to involve her scalp, cheeks, ears, neck, back, as well as membranes of the nose and vagina resulting in epistaxis and vaginal bleeding (Figure 1). This was associated with severe burning pains, and facial and peri-orbital edema. Due to the severity of her presentation, an urgent dermatology consultation was obtained, with a working diagnosis of Stevens – Johnson syndrome (Table 1). A biopsy was performed on her right forearm, and demonstrated an interface dermatitis with dermal mucin deposition (Figure 2). This pathology was consistent with a diagnosis of subacute cutaneous lupus erythematosus, which was felt to be most likely precipitated by the docetaxel exposure. Laboratory and serologic data revealed an ANA speckled pattern with a titre of 1/2560, high positive levels of anti-SSA/Ro 60 antigen at a value of 88 AU/mL (>81 AU/mL is high positive), moderately positive levels of anti-SSB/La at a value of 66 AU/mL (moderate range is 51-80 AU/mL), and elevated rheumatoid factor at 113 kU/L. A decision was made to discontinue chemotherapy after the second cycle of docetaxel, and following its discontinuation, the rash fully resolved after two months, with supportive measures including topical betamethasone cream. During her treatment course, no discernible worsening of her Sjögren’s syndrome was reported, which may have been underreported due to concurrent chemotherapy-related toxicities.

**Table 1:** **Clinical and Histological features of Stevens-Johnson syndrome (SJS) and subacute cutaneous lupus erythematosus (SCLE)**

	Stevens – Johnson syndrome (SJS)	Subacute cutaneous lupus erythematosus (SCLE)	Case commentary
Clinical features	• Mostly drug-related	• May be idiopathic or drug-induced	Although the rapidity and severity of symptom progression favored a working diagnosis of SJS, on clinical grounds it was not possible to distinguish between SJS and SCLE in the case presented
	• Characterized as dusky erythematous lesions; lesions tend to be isolated but can have confluence on the face and trunk	• Characterized as erythematous, papulosquamous or polycyclic annular plaques, typically non-scarring	
	• Typically involves the trunk, back, and extremities including palms and soles, neck, and face; often associated with painful mucosal surfaces	• Typically consists of photosensitive regions with lesions confined to sun-exposed skin (upper trunk/back, shoulders, extensor arms, neck, lateral aspects of face) with mid-facial sparing	
	• Usually occurs within 7 to 21 days after initiation of the causative drug	• Latent period between drug administration and the appearance of symptoms can range from several weeks to several years (in drug-induced SCLE)	
Histological features	• In early lesions, apoptotic keratinocytes are observed scattered in the supra-basal layers of the epidermis	• Epidermal changes and a superficial lymphocytic infiltrate in the upper dermis with apoptotic keratinocytes	Histological findings of an interface dermatitis with dermal mucin deposition is most consistent with a diagnosis of SCLE
	• In late stages, a sub-epidermal blister with overlying confluent necrosis of the entire epidermis and spare peri-vascular infiltrate composed primarily of lymphocytes is seen	• Dermal mucin deposits are commonly identified	

## Conclusions

Drug-induced SCLE is an uncommon cutaneous reaction associated with chemotherapy use. In the case presented, given the temporal relationship of the patient’s dermatological toxicities to her chemotherapy treatment, we postulate that the taxane docetaxel is the most likely culprit. In the current literature, there have been eight reported cases of SCLE associated with docetaxel, and five reported cases of SCLE associated with paclitaxel chemotherapy [1–13]. Many of the patients in these cases had a background of preceding autoimmune disease with positive anti-SSA/Ro antibodies [1–5, 8, 10, 11]. It has been suggested that the presence of serum anti-SSA/Ro antibodies may be involved in a mechanism that predisposes patients to a higher risk of developing drug-induced SCLE [4, 14]. As taxanes are hypothesized to lead to the appearance or increase in serum anti-SSA/Ro antibodies [10], the risk of drug-induced SCLE would be further heightened given a patient with pre-existing positive titres due to an autoimmune disorder. Docetaxel targets dividing cells and acts to stabilize microtubule assembly, which leads to G2/M phase cell cycle arrest followed by apoptosis [15]. Apoptotic products can then in turn incite an immunogenic response against DNA-histone and RNA-protein complexes [16]. Therefore, in an individual with a pre-existing auto-immune disorder in which antibodies against nucleoproteins already exist (such as anti-SSA/Ro), increased levels of apoptotic products from chemotherapy could stimulate an exacerbated immune response, and manifest as severe cutaneous lesions [17].

Drug-induced SCLE does not differ from idiopathic SCLE in its clinical, immunological, and histopathological characteristics [15]. Therefore, the diagnosis of drug-induced SCLE is characterized by the development of diffuse cutaneous lesions following administration of an offending agent, and its resolution upon discontinuation of the drug. In the present case, the clinical and pathological features are compatible with drug-induced SCLE, most likely secondary to docetaxel. The use of dexamethasone likely masked the true severity of the rash. Despite the addition of a course of systemic corticosteroids with prednisone with her second cycle of docetaxel, the rash rapidly progressed. The patient’s history of Sjögren’s syndrome with high levels of serum anti-SSA/Ro antibodies likely predisposed her to the development of the extensive and severe cutaneous lesions. Anti-SSA/Ro antigens have been seen to preferentially relocate to the surface of cultured keratinocytes that had been irradiated with ultraviolet radiation [18]. It should be noted that her skin lesions were distributed over photo-exposed areas of her body, suggesting that induction of photosensitivity may have been a contributing mechanism to the development of her skin eruptions. Lastly, the patient also had a history of chronic benign neutropenia, and was given peg-filgrastim with her chemotherapy cycles to support her white blood cell and neutrophil counts. The increased number of circulating white blood cells and neutrophils may also have contributed to a heightened immune response, further exacerbating her cutaneous reaction to the docetaxel.

Although SCLE is classically characterized as either idiopathic or drug-induced, there has been sufficient evidence in the literature to consider certain cases of SCLE to have a paraneoplastic etiology [19]. There have been multiple reports of SCLE arising in the setting of internal malignancy including two cases in breast adenocarcinomas [20]. Like other paraneoplastic dermatoses, presentations of paraneoplastic SCLE follow the criteria in that the cutaneous findings appear temporally after development of the malignancy, and their clinical courses typically run parallel [21]. As such, paraneoplastic SCLE tends to resolve after successful treatment of the malignancy. However, because of the role of chemotherapy treatments in causing tumor regression and the timing of its administration, it may sometimes be difficult to differentiate drug-induced SCLE from paraneoplastic SCLE. This is especially true if the appearance of skin eruptions occurs shortly after beginning systemic chemotherapy. However, if the lesions regress quickly after discontinuing chemotherapy, as was observed in this case, then the etiology would be most compatible with drug-induced SCLE. Regardless, it is important to also consider paraneoplastic SCLE in the differential diagnosis.

Drug-induced subacute cutaneous lupus erythematosus (SCLE) is an uncommon but potentially severe disease state associated with docetaxel chemotherapy. The clinician needs to recognize and consider this entity in the differential diagnosis of chemotherapy-associated cutaneous reactions, given its severity, and the importance of discontinuing the offending agent as primary management. Although the exact mechanisms behind drug-induced SCLE remain unclear, patients with a pre-existing autoimmune disorder appear to have a higher incidence when exposed to offending agents including chemotherapy. The identification and discontinuation of the offending agent is essential in the management of drug-induced autoimmune reactions.

## Consent

Written informed consent was obtained from the patient for the publication of this case report and any accompanying images. A copy of the written consent is available for review by the Editor-in-Chief of this journal.

## Abbreviations

SCLE: Subacute cutaneous lupus erythematosus
FEC-D: 5-fluorouracil, epirubicin, cyclophosphamide, docetaxel chemotherapy
G-CSF: Granulocyte colony-stimulating factor
ANA: Anti-nuclear antibody
DNA: Deoxyribonucleic acid
RNA: Ribonucleic acid.

## References

[1] Callen J (2013). Drug-induced subacute cutaneous lupus erythematosus – filling the gap in knowledge. JAMA Dermatol.

[2] Chen M, Crowson A, Woofter M, Luca M, Magro C (2004). Docetaxel (Taxotere) induced subacute cutaneous lupus erythematosus: report of 4 cases. J Rheumatol.

[3] Lebeau S, Tambe S, Sallam M, Alhowaish A, Tschanz C, Masouye I, Borradori L (2013). Docetaxel-induced relapse of subacute cutaneous lupus erythematosus and chilblain lupus. J Dtsch Dermatol Ges.

[4] Adachi A, Horikawa T (2007). Paclitaxel-induced cutaneous lupus erythematosus in patients with serum anti-SSA/Ro antibody. J Dermatol.

[5] Dasanu C, Alexandrescu D (2008). Systemic lupus erythematosus associated with paclitaxel use in the treatment of ovarian cancer. South Med J.

[6] Funke A, Kulp-Shorten C, Callen J (2010). Subacute cutaneous lupus erythematosus exacerbated or induced by chemotherapy. JAMA Dermatol.

[7] Gantzer A, Regnier S, Cosnes A, Ortonne N, Wolkenstein P, Bagot M, Duong T (2011). Subacute cutaneous lupus erythematosus and cancer: two cases and literature review. Annales de Dermatologieet de Venereologie.

[8] Lamond N, Younis T, Purdy K, Dorreen M (2013). Drug-induced subacute cutaneous lupus erythematosus associated with nab – paclitaxel therapy. Curr Oncol.

[9] Lortholary A, Cary-Ten Have Dallinga M, El Kouri C, Morineau N, Ramee J (2007). Paclitaxel-induced lupus. Presse Med.

[10] Marchetti M, Noland M, Dillon P, Greer K (2013). Taxane associated subacute cutaneous lupus erythematosus. Dematol Online J.

[11] Pham A, Berz D, Karwan P, Colvin G (2011). Cremophor-induced lupus erythematosus-like reaction with taxol administration: a case report and review of the literature. Case Rep Oncol.

[12] Renner R, Sticherling M (2008). Incidental cases of subacute cutaneous lupus erythematosus in association with malignancy. Eur J Dermatol.

[13] Vihinen P, Paija O, Kivisaari A, Koulu L, Aho H (2011). Cutaneous lupus erythematosusafter treatment with paclitaxel and bevacizumab for metastatic breast cancer: a case report. J Med Case Rep.

[14] Srivastava M, Rencic A, Diglio G, Santana H, Bonitz P, Watson R, Ha E, Anhalt G, Provost T, Nousari C (2003). Drug induced, Ro/SSA-positive cutaneous lupus erythematosus. Arch Dermatol.

[15] Lowe G, Henderson C, Hansen C, Sontheimer R (2011). A systematic review of drug-induced subacute cutaneous lupus erythematosus. Brit J Dermatol.

[16] Stollar B, Stephenson F (2002). Apoptosis and nucleosomes. Lupus.

[17] Rahman A, Stollar B (2008). Origin and structure of autoantibodies and antigens in autoimmune rheumatic diseases. Lupus.

[18] Igarashi T, Itoh Y, Fukunaga Y, Yamamoto M (1995). Stress-induced cell surface expression and antigenic alteration of the Ro/SSA autoantigen. Autoimmunity.

[19] Evans KG, Heymann WR (2013). Paraneoplasticsubacute cutaneous lupus erythematosus: an underrecognized entity. Cutis.

[20] Shaheen B, Milne G, Shaffrali F (2009). Subacute cutaneous lupus erythematosus associated with breast carcinoma. Clin Exp Dermatol.

[21] McLean D (1986). Cutaneous paraneoplastic syndromes. Arch Dermatol.

